# A standing wave tube-like setup designed for tomographic imaging of the sound-induced motion patterns in fish hearing structures

**DOI:** 10.1186/s12915-025-02388-4

**Published:** 2025-09-15

**Authors:** Isabelle P. Maiditsch, Tanja Schulz-Mirbach, Martin Heß, Friedrich Ladich, Marco Stampanoni, Christian M. Schlepütz

**Affiliations:** 1 Faculty of Biology, Zoology, Ludwig-Maximilians-University, Planegg-Martinsried, Germany; 2 Swiss Light Source, Paul Scherrer Institute, Villigen, Switzerland; 3 Department of Behavioral and Cognitive Biology, University of Vienna, Vienna, Austria; 4 Institute for Biomedical Engineering, University and ETH Zürich, Zurich, Switzerland

**Keywords:** Acoustic field, Sound pressure, Particle motion, Test tank, High-resolution imaging

## Abstract

**Background:**

Modern bony fishes exhibit a considerable variation in the morphology of their hearing structures, and the morphological composition of these has been studied for centuries. However, the precise interaction and contribution of individual structures to hearing remains unclear in many species. Measurements of their motion in situ are challenging and pose the risk of damage or altering results through invasive intervention. Recent developments in time-resolved synchrotron-radiation-based tomography have opened up possibilities for non-destructive quantification of the micron-level motion patterns of the auditory system. However, the strict requirements for miniaturised acoustic environments compatible with tomographic imaging hinder the production of ideal and well-characterised sound fields. To address this issue, we present the design of a miniature standing wave tube-like setup equipped with the necessary sensors to tune and monitor the sound field in situ, thereby generating and recording the desired acoustic conditions during experiments.

**Results:**

By incorporating hydrophones into the tube of the standing-wave setup, we achieved a precise adjustment of the acoustic field within the tube at various frequencies. We generated and measured frequencies up to 2 kHz that fall within the relevant hearing spectrum of otophysan fish. The setup allows for the determination and adjustment of sound pressure levels during tomographic measurements, and phases can be regulated to achieve distinct differences between maximum (0° phase shift) and minimum (180° phase shift) sound pressure at the centre of the test tube.

**Conclusions:**

We are able to visualise the motion of the peripheral auditory structures from the swim bladder to the Weberian ossicles and the otoliths (sagittae) in terms of maximum and minimum (sound-induced particle motion) sound pressure, respectively. This methodology has been successfully applied to various otophysan fish species and is demonstrated in the example of a glass catfish (*Kryptopterus vitreolus*). Our setup not only enhances our understanding of basic principles in fish bioacoustics but also sets a new standard for non-invasive, high-resolution imaging techniques in the field of aquatic sensory biology.

**Supplementary Information:**

The online version contains supplementary material available at 10.1186/s12915-025-02388-4.

## Background

Fish auditory structures exhibit a huge diversity across different species [1]. Many basic questions about the interaction and contribution of these individual structures to the hearing mechanism and their impact on hearing abilities remain unanswered [2]. For instance, the impact of varying shapes and dimensions of the otoliths, the Weberian ossicles, and the swim bladder on otolith motion remains largely unknown. Reconciling the available knowledge into a coherent model poses a considerable challenge, mainly due to the complexity of observing the hearing systems in action under representative physiological conditions. Untested hypotheses, often formulated decades ago, highlight our fundamental lack of understanding of the basic mechanisms involved [3, 4]. Various methods to study fish auditory structures have evolved since the nineteenth century, beginning with the seminal anatomical work of Weber [5, 6] and Retzius [7], and continued with experiments by von Frisch on the auditory systems in otophysans [8, 9]. Subsequent research has provided deeper insights into the morphology and function of inner ears and related organs, as well as the hearing abilities of different fish species [10–12]. Despite significant advances in fish hearing research, especially in the twenty-first century [13–19], the link between auditory organ complexity and hearing performance remains unclear [20].

Two distinct issues must be addressed to study the motion patterns of fish auditory structures in their native condition. First, the structures need to be analysed using a non-invasive technique, preserving the structures in their native anatomical condition with sufficient spatial and temporal resolution to reveal the relevant dynamics. Secondly, the motion pattern should be measured within a well-defined sound field. Ideally, the sound-induced in situ movements of fish auditory structures are studied within the test subject itself without their response being influenced due to surgical procedures or tissue modifications.

Former experimental approaches have failed to non-destructively investigate the effects of sound on fish hearing structures in real-time [21, 22]. Novel Synchrotron radiation-based imaging techniques enable the visualisation and quantification of internal moving structures at a high spatial resolution of a few micrometres and a temporal resolution of up to 3 kHz in a non-invasive way [23–26], allowing the preservation of the natural biomechanical properties and response of the fish. This technique was demonstrated by studies at the European Synchrotron Radiation Facility (ESRF, Grenoble, France) [27, 28] and the Swiss Light Source (SLS, Paul Scherrer Institute, Villigen, Switzerland) [23]. However, real-time observation imposes specific limitations on the setup when combining acoustic field control with tomographic imaging. X-ray imaging requires miniaturising the tank cross sections to reduce X-ray absorption by the sample environment. In the case of tomographic imaging, it requires rotating the setup through 180° without obstructing X-ray exposure. The original designs by Schulz-Mirbach et al. [28] and Maiditsch et al. [23] were based on Hawkins and MacLennan [29], who developed a standing wave tube for an optimal acoustic field in hearing measurements. Their design featured thick steel walls to ensure rigidity and minimise radial water movements. This tank design is unsuitable for tomographic imaging, so the material was replaced with transparent Plexiglas® tubes, and the tank size and weight were reduced. The 2020 setup [28] for radiographic imaging was lighter and smaller compared to Hawkins and MacLennan, but still featured thick tube walls and a relatively large water volume (1.7 L), resulting in significant X-ray absorption, thus complicating imaging. In 2022 [23], we further miniaturised the standing-wave tube-like setup to meet the conditions to perform fast tomographic imaging at the SLS. By using a smaller tube diameter and thinner tube walls (volume 49.9 ml), the weight and size of the setup were reduced even more, minimising X-ray absorption by both the tube walls and the water body. Additionally, positioning the tube and specimen upright allowed for a rotation of at least 180° and the unobstructed illumination of the sample with X-rays from all directions.

Meeting imaging requirements was necessary, but the miniaturisation and change of material of the setup impaired control of the acoustic field in the experimental tank. Achieving ideal acoustic conditions in the setup of Maiditsch et al. [23] has proven difficult, and reproducibility remains limited. Building on the work of Hawkins and MacLennan [29] we adapt the previous design to achieve a more active control over the effective sound field during experiments, allowing for a better adjustment of sound pressure levels and separation of phases as fare as possible (maximum 0° in-phase and minimum 180° out-of-phase sound pressure within our tube), across a broader frequency spectrum. We accept non-ideal acoustic properties and equip the system with diagnostics to control and monitor sound fields for desired experimental conditions. This was achieved by (a) integrating two hydrophones into the imaging setup for direct monitoring of the sound levels during experiments and (b) the construction of a geometrically identical test tube featuring additional hydrophone measurement ports for prior or subsequent ex situ cross-validation of the sound field.

This paper describes the design, construction and operation of our modified acoustic tank and provides detailed characterisations of its performance. The success of the improved design is corroborated by experimental X-ray imaging results of dynamic motion patterns observed in the auditory structures of a glass catfish (*Kryptopterus vitreolus*) under alternating minimum and maximum sound pressure stimulation. This setup deepens our understanding of fish bioacoustics by allowing direct observation of auditory structures. It establishes a benchmark for non-invasive, high-resolution aquatic sensory imaging methods in biology. Therefore, our work aims to provide readers with all the necessary information to understand and replicate our experimental setup, as well as to adapt the concept to other novel experimental capabilities.

## Methods

### Design principles for the new acoustic setup

The previous tank design utilised by Maiditsch et al. [23] proved to be well-suited with regard to the X-ray imaging requirements. It allowed for unobstructed X-ray exposure of the sample, yielding excellent data quality. The main issue is the lack of control and feedback on acoustic properties, as the tank’s shape and material are not optimal for ideal standing wave sound fields. However, since these aspects are tailored to X-ray imaging, improvement options are limited. Instead, the approach we choose in this work is to accept the non-ideal acoustic properties and to equip the system with the necessary diagnostics to actively control and monitor the sound fields to achieve the desired experimental conditions.

The aim is to measure the sound field characteristics at the specimen’s imaging position in the acoustic tank. However, the sample occupies this position during X-ray measurements, preventing the placement of additional sensors in the horizontal tomographic imaging plane. We thus take a two-step approach to address this issue. First, we equip the experimental tank for X-ray imaging (referred to as “Img-Tank”) with two reference hydrophones positioned above and below the imaging plane, allowing us to monitor the corresponding sound pressure levels during imaging experiments. Secondly, we build a geometrically identical replica of the Img-Tank, enabling the addition of sensors, e.g. in the location of the sample, for independent tests without X-ray beam exposure (referred to as “Test-Tank”). The Test-Tank can be used to determine a suitable set of sound generation settings prior to imaging experiments or to verify the effect of a particular setting at the location of the sample after imaging.

### Design details

**Fig. 1:**
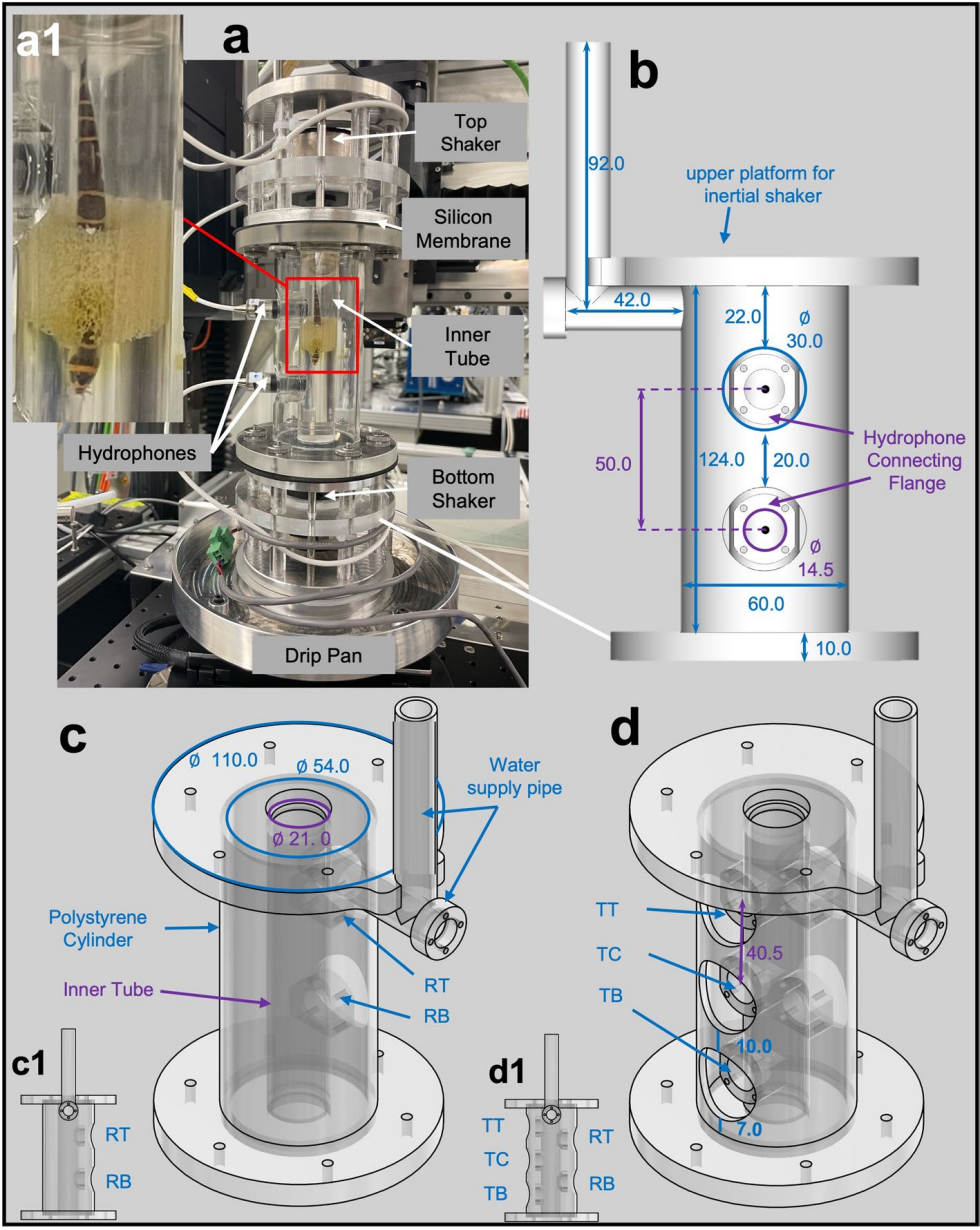
Overview of the two-shaker standing-wave tube setup. **a** shows an image of the two-shaker standing-wave tube-like setup for imaging experiments (Img-Tank) with two hydrophones flange openings with a test subject placed in the centre of the tube (**a1**) using a piece of porous foam wrapped around the fish. **b** and **c** show schematics of Img-Tank depicting the tank dimensions and parts (inertial shakers not shown), **b** with the centre of the hydrophone marked with a black dot and (**c1**) a schematic side-view of the tank showing the disposition of the two hydrophone openings on the right side (RT and RB). **d** show schematic drawing of the Test standing-wave tube-like setup (Test-Tank) with five openings and (**d1**) a schematic side-view of the tank showing the partition of the five openings on both sides (RT, RB, TT, TC, TB). **b**–**d** values are given in millimetres; colour code: blue—labelling of dimensions and parts for the outer tube, purple—labelling of dimensions and parts for the inner tube; ø—diameter

The basic tank geometry is shown in Fig. 1 and consists of a vertical Plexiglas® tube that can be filled with water and hosts the fish specimen in its centre, in the case of the Img-Tank (Fig. 1a). At the top and bottom end of the tube, two inertial shakers transmit sound waves to the water body via a flexible silicone membrane sealing the volume. To support the weight of the upper shaker and to provide sufficient structural integrity and stiffness to the entire setup, the inner water-filled tube is surrounded by a larger outer Plexiglas® tube. Both tubes feature several openings for the installation of hydrophones.

The inner Plexiglas® tube has an inner diameter of 21 mm and a wall thickness of 2 mm, while the supporting outer cylinder measures 54 mm in inner diameter at a wall thickness of 3 mm. They are glued into circular 10 mm thick round plates with 110 mm diameter at the top and the bottom. The entire structure has a height of 144 mm, resulting in a total volume of the water-filled chamber of 49.9 ml. Below the upper end plate, a water supply pipe leads to the inner chamber for filling or draining the water volume. It can be sealed with a plug that is shaped to be flush with the inner chamber surface to maintain the cylindrical geometry of the tube.

The main new features of the tanks are the lateral openings, located at 90° from the water supply line, to accommodate hydrophones during the measurements. Both tank versions (Img-Tank and Test-Tank) are equipped on one side with a pair of openings above and below the imaging location to place two mini-hydrophones (Brüel & Kjær 8103, sensitivity: − 211 dB re. 1 V μPa − 1; diameter: 9.5 mm). We will refer to these locations as the reference position top (RT) and reference position bottom (RB), respectively. The openings have a 14.5-mm diameter through the inner tube wall and include an adapter flange for connecting a hydrophone adapter or sealing plug. All flanges are equipped with a sealing O-ring and four M3 threaded holes to secure the fittings with screws. The outer tube contains a 30 mm diameter hole coaxial to the one on the inner tube for easy access to the mounting flange and exchange of the adapters. In the presence of these flanges and the outer tube holes, there remains a 20-mm-tall section around the middle of the tube that is totally unobstructed by auxiliary attachments and maintains the perfect rotational symmetry of the Img-Tank.

**Fig. 2:**
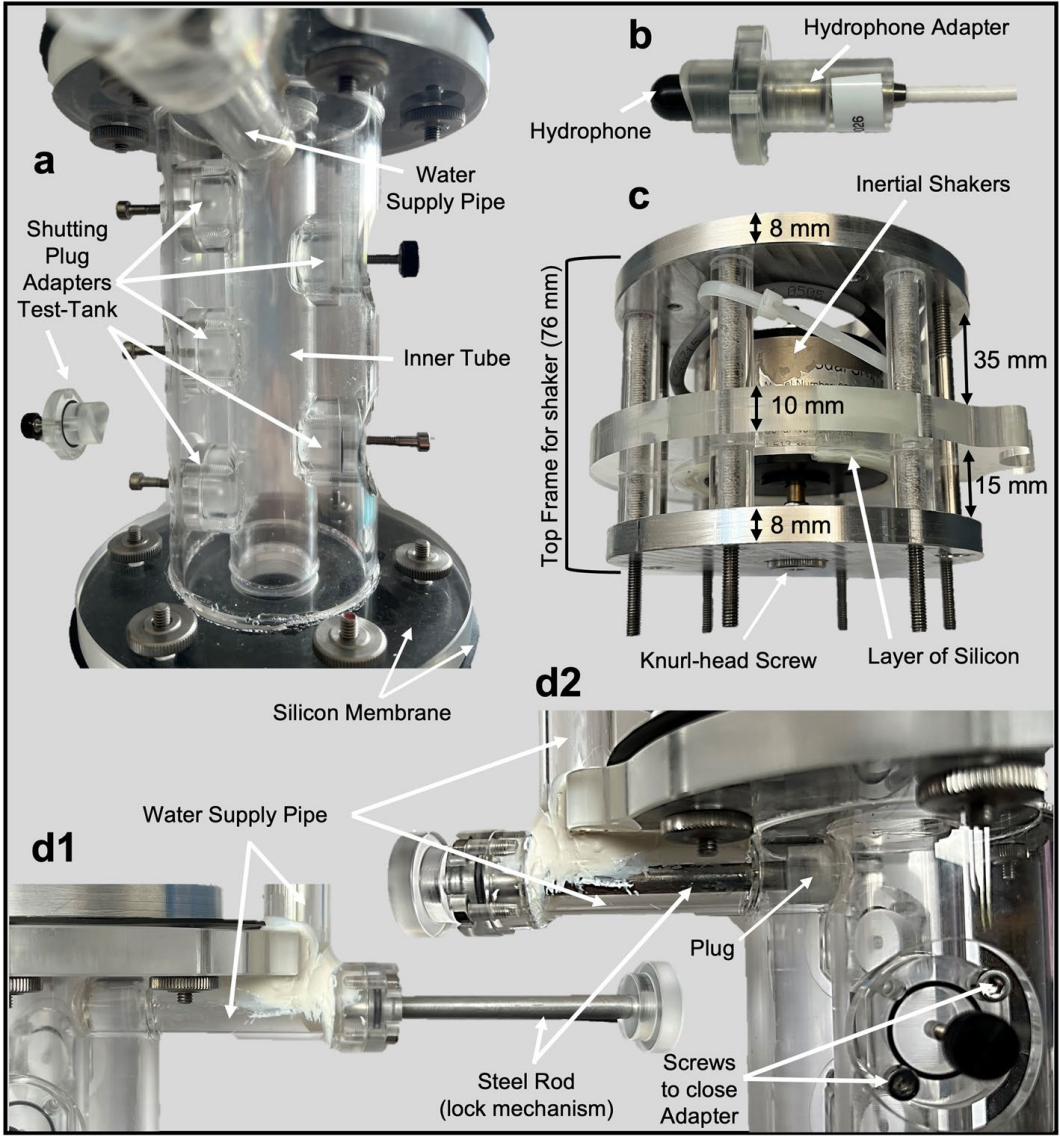
Overview of various parts of the two-shaker standing wave tube setup. **a** show a close-up of the Test-Tank with five hydrophone openings, sealed with Plexiglas® plugs which are mounted with screws (**d2**). **b** shows a mini hydrophone mounted in an adapter. **c** shows the preassembled frame in which the shakers are fixed with a layer of soft silicon; the shakers are equipped with a knurl-head screw that transmits the vibrations to the water. This installation is directly attached to the tubes, in this case, to the top. **d1** shows the steel rod with a Plexiglas® plug at the end, which is inserted through the horizontal part of the supply pipe (**d2**)

The Test-Tank (Figs. 1d and 2a) features three additional openings with the same dimensions on the side opposite to the reference openings that can be used to place hydrophones within the water volume. One opening is positioned exactly at the centre of the tube (TC), giving access to the nominal imaging position of the fish specimen. Two more openings are placed as close as feasible to the top and bottom shakers (referred to as TT and TB, respectively) to separate the individual contributions of each sound source on the generated sound pattern more easily.

Inertial shakers (2002E, PCB Synotec) are mounted within a bespoke frame that can be easily and quickly attached to the tank bodies via six screws through the tank’s end plates. The frame itself has a total height of 76 mm and consists of three round plates of 100 (aluminium) or 110 mm (Plexiglas®) diameter. Those are separated by six pairs of Plexiglas® spacers through which the six threaded attachment-rods are running (Fig. 2c). The rear aluminium plate (facing away from the vibrating end of the shaker) holds screw holes for mounting the rig onto the imaging sample holder. The front aluminium plate (facing the water tank) has a central opening for the shaker’s vibrating end to extend towards the tank. The shaker itself is attached only to the central plate made from 10 mm thick Plexiglas®. The shaker is glued into a central aperture (radius 32.5 mm), which is approximately 7 mm larger than the shaker body radius (25.4 mm), using a layer of soft silicone to minimise the transmission of vibrations to the rigid frame and the rest of the setup. A 0.5-mm silicone membrane seals the inner acoustic tank and connects to the shakers by clamping between the shaker frame’s front plate and the acoustic tank’s end plates. The shakers’ vibrating ends are terminated with a knurl-head screw (Fig. 2c; diameter: 16 mm, thickness: 3 mm), which pushes against the silicone membrane and transmits the generated sound stimuli into the water. The amount of pre-tension that the knurl-head screws apply to the silicone membrane can be manually adjusted using the attachment screw to the shaker.

The mini-hydrophones used during the experiments are pre-fitted into dedicated adapters, providing a tight seal between the adapter material and the hydrophone body via their inbuilt O-rings and then secured to the respective opening flanges (Fig. 2b). The hydrophone can be flexibly positioned due to its elongated shape, allowing radial movement between the adapter and hydrophone. This adjustment can range from nearly fully crossing the tube diameter to complete retraction behind the wall surface. Those flanges not used for hydrophones can be sealed with plugs, allowing for flexible configurations regarding the number and locations of hydrophones used simultaneously (Fig. 2a). All plugs are designed such that their surface perfectly follows the inner wall of the chamber. To protect the high-tech and electronic setup in the beamline from unlikely spillage of water, the entire two-shaker setup is mounted on a circular drip pan during the experiments (Fig. 1a).

The full set of technical drawings of our acoustic tank setup has been published as PDF and stp-files for open access online on Zenodo (10.5281/zenodo.16737258) [30]. All Plexiglas® parts have been manufactured and assembled by ACRYLINE AG (8154 Oberglatt, Switzerland).

### Tank assembly and sample preparation

The assembly of the tank setups for all experiments followed a strict, bottom-to-top series of steps to ensure reproducibility and comparability between experiments. Starting with the bottom shaker frame standing on its back plate, the lower silicone sealing membrane was placed over the front plate, covering the central aperture. Then the Plexiglas® tank was mounted on top, secured tightly against the silicone to ensure a good seal using six knurl-head nuts on the screws from the shaker frame. Two hydrophones were then inserted and fixed into the corresponding reference openings at RT and RB to seal the tube from the sides. They were not removed thereafter for the full duration of an experimental campaign. We also ensured that the same hydrophones were consistently placed in the same openings for all experiments by identifying them by their serial numbers (position RT—serial number 3342115; position RB—serial number 3139026). The hydrophones were inserted just enough so that the front end of each hydrophone was levelled with the tube wall, ensuring they did not protrude into the water column and allowing sound waves to reach the test object without obstruction.

With the lower shaker secured and the hydrophones fixed in the flange openings such that the tube was effectively sealed, the tank is now ready to be filled with water through the upper central opening. First, both the water and the foam, used to affix the fish within the tube, were degassed in a vacuum oven (MODLE) for at least one hour, or until no visible air bubbles remained. We filled the inner cylinder with degassed and demineralised water to the level of RT, to prevent water overflow when inserting the test object and to ensure that the test object would be fully submerged once inside the tube. Second, the test object (fish), which had been prepared in advance (for detailed handling procedures, see Schulz-Mirbach et al. [28] and Maiditsch et al. [23]), was wrapped in wet degassed foam and gently inserted headfirst into the tube, with the head or the area of interest positioned precisely at the centre of the tube (Fig. 1, a1). Thereafter, the tube was completely filled with water until slightly overflowing through the upper central opening, and likewise, the supply pipe filled up with water. Afterwards, the upper shaker frame, with the silicone sealing membrane placed over the front plate, was attached to the top platform and secured with screws. Once fully assembled, the inner tube was checked for the presence of any remaining trapped air bubbles, which would then be released by the open water supply pipe (Fig. 2, d1 and d2) and tilting the entire apparatus with the supply pipe pointing upwards. After complete air removal, the plug of the water supply line was closed (Fig. 2, d2). The setup was then mounted on the sample manipulator within the beamline. The procedure for the Test-Tank was identical, the only differences being that no test objects were involved and the placement of hydrophones in the additional openings as required. We ensured that the same hydrophones were consistently allocated to the same shaker for all experiments conducted. Thus, the hydrophone at RT was used at TT, while the one for RB was used at TB. Additionally, a third hydrophone was implemented only for the central position (position TC—serial number 3342119). All measurements within the Test-Tank were conducted in a laboratory setting without the presence of an X-ray beam.

### Sound stimulation

**Fig. 3:**
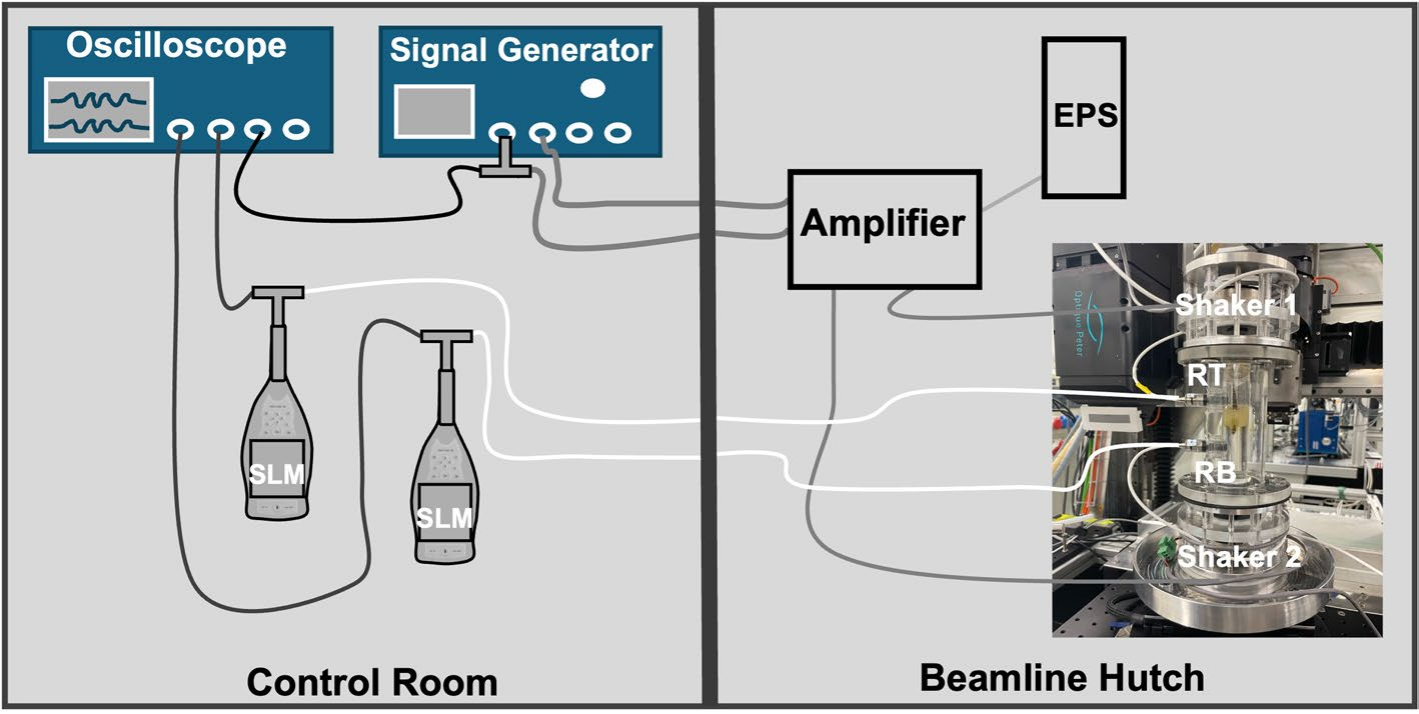
Schematic overview of the acoustic setup. The control room (left) and the beamline hutch (right). EPS external power supply, RT/RB hydrophone at the top/bottom reference position, SLM sound level meter

The electronics setup used to create the acoustic stimulus is shown in Fig. 3. The electronic sine wave signals to generate the sound stimuli were produced by a signal generator (SiG; Tektronix, AFG 3102) and transmitted to the shakers after passing through an amplifier (AMP; S.M.S.L. SA 36 A Pro, Shenzhen ShuangMuSanLin Electronic Co.). To avoid power hum, the amplifier was powered through an external isolated power supply (EPS; EA-PSI 8032–10, TOMCAT). Signals for the upper and lower shaker were generated independently using two channels of the SiG and individually amplified through the stereo inputs/outputs of the amplifier. Additionally, the generated stimulus signals were passed to an oscilloscope (OSC; Tektronix TBS 2000B Series, TBS2074B) for visualisation. The hydrophones to record the sound conditions in the water volume were connected to sound level meters (SLM; Brüel & Kjær 2250 and 2250-L-S) as well as to the oscilloscope to verify the relative amplitudes and phases between the signals. Both hydrophones were calibrated prior to experiments with a hydrophone calibrator (Brüel & Kjær 4229).

Only for the imaging experiments, the setup was strategically divided between two separate areas (Fig. 3). The standing-wave tube-like setup with its two hydrophones (Img-Tank), the amplifier and the external power supply were located inside the experimental hutch, an area inaccessible during the measurements due to X-ray radiation. Amplifier settings were kept unchanged throughout the experiments. The remaining control components (SiG, SLMs, OSC) were placed in the control room, facilitating real-time monitoring and precise control of the acoustic environment throughout the experimental procedure.

Stimuli were tested and measured at frequencies of 450, 550, 700, 1250, 1500, 1750, and 2000 Hz at maximum sound pressure (the shakers driven 0° in-phase) and at 450, 550, 700, 1250, and 1500 Hz at minimum sound pressure (the shakers driven 180° out-of-phase). These frequencies fall within the hearing range of the tested fish species [18]. Before starting the imaging measurements for a given acoustic condition, SPLs of the two shakers needed to be adjusted separately, using a hydrophone at RT for the top shaker and at RB for the bottom shaker, to ensure that SPLs at those two positions matched. The SPLs were controlled directly via the signal generator by adjusting the amplitude output, given in peak-to-peak voltage (Vpp). First, the desired frequency was played through the upper shaker, which was controlled through the first SiG output channel, and Vpp was adjusted to produce the desired SPL measured at RT while the lower shaker remained off. Then, the same procedure was repeated for the lower shaker, controlled via the second SiG channel and measured at RB, while the upper shaker was switched off. The SPLs were chosen based on preliminary tests, the type of experiment, and the species tested, with the intention of being as low as possible while ensuring the motion of the structures remained visible. In some cases, the SPLs were later adjusted during the experiment to optimise the motion of the targeted structures, either by increasing or decreasing the signal amplitudes. Consequently, not all experiments used the same SPLs, but efforts were made to minimise variability.

### Sound field characterisation

Before conducting actual tomographic microscopy experiments, preliminary tests in the lab were used to determine the intrinsic characteristics of the new setups. First, acoustic measurements were conducted with both tanks to confirm that the response, as measured by the two reference hydrophones, to stimulation settings at different frequencies and SPLs in the Img-Tank could be replicated in the Test-Tank, and vice versa. In a next step, using the Test-Tank with a third hydrophone placed at TC, a set of reasonable stimulation amplitudes and corresponding reference hydrophone responses were determined as a function of frequency to yield the desired SPLs at the sample location. Further experiments were conducted using all five openings in the Test-Tank, to better assess the tank’s acoustic properties and validate the optimal positioning of the two reference openings used in the Img-Tank (Fig. 1 d1: RT and RB).

Based on the established equivalence of the two tanks’ acoustic properties, it was also possible to perform retroactive acoustic measurements in the Test-Tank under the same conditions used in the Img-Tank during experiments. Utilising the third hydrophone installed at TC of the Test-Tank and adjusting the hydrophone responses at RT and RB to the values reported during the imaging experiments, SPLs at the specimen location were determined. Ambient noise levels in the beamline experimental hutch were measured before and after the imaging experiments using the two miniature hydrophones within the Img-Tank and the SLM.

### X-ray tomographic imaging

All experiments and X-ray tomographic microscopy measurements were carried out at the TOMCAT beamline X02DA of the Swiss Light Source (SLS) located at the Paul Scherrer Institute (PSI) in Villigen, Switzerland [31]. For a detailed description of the imaging process, one is referred to the imaging protocol and acquisition parameters studies by Lovric et al. [32, 33] A detailed description of the data collection procedure can be found in Maiditsch et al. [23]. For the data presented in this paper, we used the polychromatic X-ray beam from the bending magnet source, filtered with 5 mm of glassy carbon and 4 mm of borosilicate glass, resulting in a detected peak energy around 25 keV. X-ray images were converted to visible light by a 150 µm thick LuAG:Ce scintillator and collected using a 4 × magnification high numerical aperture optical microscope in conjunction with the GigaFRoST camera, with an effective pixel size of 2.75 µm at full frame rates up to 1.25 kHz. Using a retrospectively gated data acquisition scheme and exposure times of 0.14286 ms, we achieved an effective temporal resolution of 15 kHz for the measurements presented in this work.

### Different sound conditions within the tube

To visually demonstrate the proper functioning of the tube and the control of the acoustics at the centre of the tube, a 2D dynamic radiography of a glass catfish (*Kryptopterus vitreolus*) was recorded while subjecting the specimen to a beating pattern, which was produced by applying pure tones of 700 Hz to the top shaker and 736 Hz to the bottom shaker, resulting in a beating frequency of 36 Hz. This beating pattern manifests itself as a periodic variation in the relative phase and, consequently, amplitude at the test subject, resulting in alternating maximum sound pressure (constructive interference, in-phase 0°) or minimum sound pressure (destructive interference, out-of-phase 180°) conditions every 27.8 ms. The collected imaging data was retrospectively gated with respect to the reference frequency (700 Hz) and the relative phase between stimuli to yield the time-averaged response of the fish specimen to the beating sound field. Fiji software [34] was employed to visualise the motion of each individual hearing structure of the glass catfish.

## Results

### Performance of the standing wave tube-like tanks

**Fig. 4:**
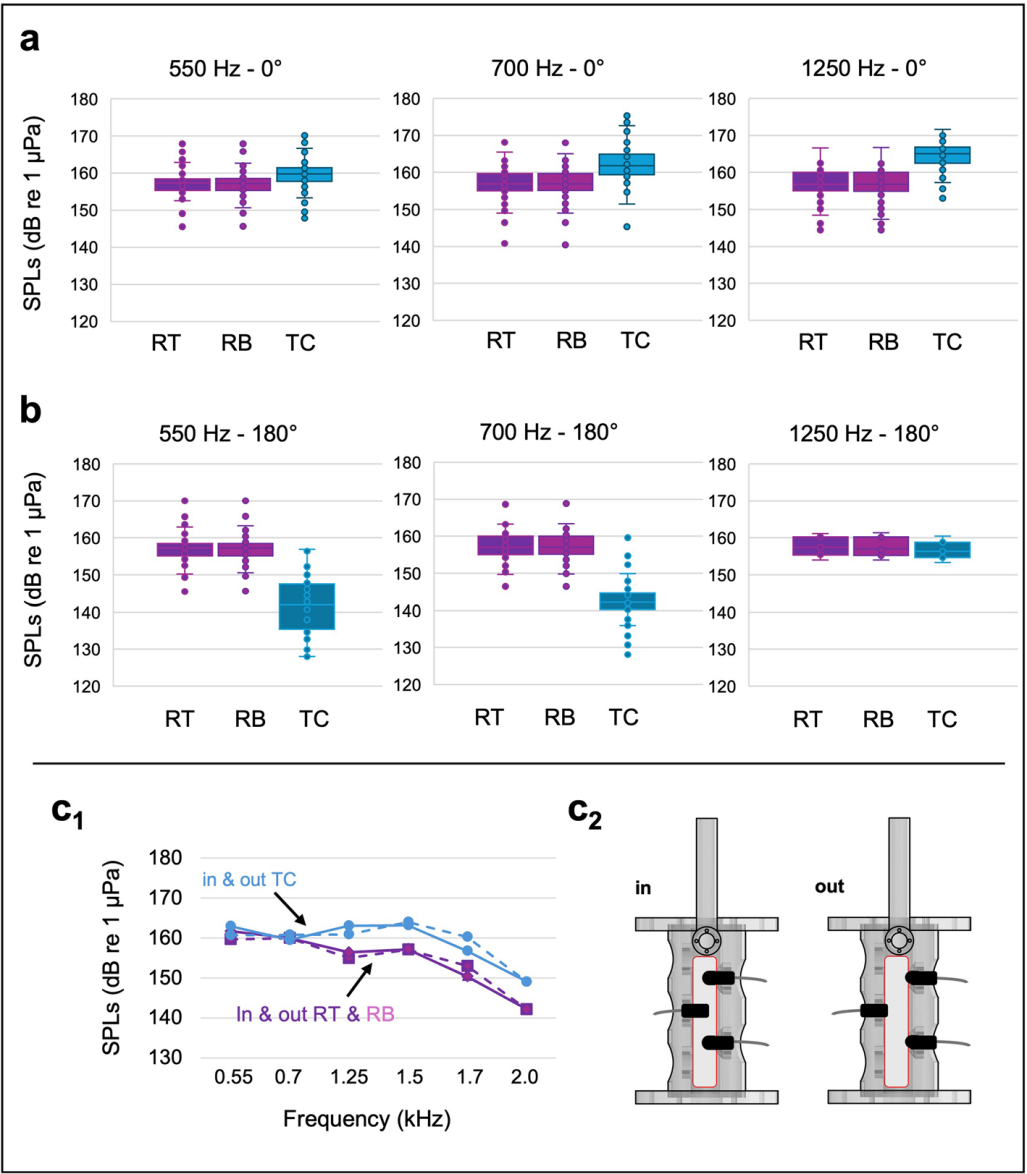
Differences of sound pressure levels (SPLs) determined at different frequencies. Box plot of SPLs measured at position RT (near upper shaker, colour code: dark purple), RB (near lower shaker, colour code pink) and at the centre of the tube TC (colour code blue) during **a** the in-phase (0°) and **b** the out-of-phase condition (180°) at three test frequencies. **c**_**1**_ shows the comparison of SPLs where hydrophones (RT, RB, TC) were placed either in the centre of the water body of the tube (in: blue and purple solid lines) or, as in the imaging experiments, flush with the wall (out: blue and purple dashed lines). **c**_**2**_ shows the schematic images of the hydrophone placement

Our experiments demonstrate that the shakers can be precisely adjusted using nearby hydrophones, enabling accurate adjustment of SPLs. Tests conducted prior to the imaging experiments indicated that both shakers need to generate matching SPLs, with a maximum variation of 0.7 dB between them, to achieve the desired separation between maximum and minimum sound pressure at the centre of the inner tube. However, when the signal generator was configured to output identical signal amplitudes (Vpp) for both shakers, the measured SPLs varied between 2.2 and 10.9 dB (see Additional File 1: Sheet1 and Sheet2). This effect was observed both in the Img-Tank and the Test-Tank. The exact origin of this discrepancy is not precisely known but may result from a slightly different response of the two shakers, due to differences in the suspension systems and associated static forces, as well as non-reproducible differences in the coupling efficiency and sound transmission between the shaker and the water body. Regardless of its cause, this difference corroborates the need for a separate adjustment of the two shaker signals. Due to this active tuning, no considerable differences were observed in the SPLs recorded between the two reference positions (RT and RB) across all frequencies and both phases during the imaging experiments (Fig. 4; Additional File 1: Sheet1). Shakers were adjusted to achieve consistent SPLs with a mean difference between 0.0 dB and 0.3 dB (Table 1).

**Table 1:** Mean values (± S.E.) of sound pressure levels collected during experiments. In total, 571 experiments were conducted, and SPLs at each frequency were recorded under different conditions (phase: 0° versus 180°) and at various locations (RT, RB, and TC). The mean SPL and its standard error (in parentheses) for each frequency, phase (0° and 180°) and position (RT, RB and TC) are shown. The mean difference of SPLs between positions RT and RB is provided for all frequencies and phases, with the maximum difference over all trials shown in parentheses. All SPLs are given in dB re 1 μPa

Frequency (Hz)	No. of exp	Phase°	Img-Tank RT	Img-Tank RB	mean diff (max. diff.) RT and RB	Test-Tank TC
A.N. Beamline			118.0	118.5		
450	9	0	158.3 (± 1.7)	158.3 (± 1.7)	0.1 (0.3)	162.4 (± 1.8)
550	119	0	156.7 (± 0.3)	156.8 (± 0.3)	0.1 (0.5)	159.4 (± 0.4)
700	116	0	155.5 (± 0.4)	155.6 (± 0.4)	0.1 (0.6)	161.0 (± 0.4)
1250	74	0	156.8 (± 0.5)	156.7 (± 0.5)	0.1 (0.6)	164.2 (± 0.5)
1500	49	0	155.2 (± 0.8)	155.2 (± 0.8)	0.1 (0.6)	163.9 (± 0.7)
1750	26	0	149.5 (± 1.2)	149.5 (± 1.2)	0.1 (0.3)	155.5 (± 1.1)
2000	3	0	142.9 (± 2.1)	142.9 (± 2.0)	0.0 (0.1)	149.4 (± 2.3)
450	3	180	154.9 (± 4.1)	154.6 (± 4.2)	0.3 (0.4)	147.1 (± 3.9)
550	73	180	156.9 (± 0.4)	157.0 (± 0.4)	0.1 (0.5)	141.1 (± 1.1)
700	90	180	157.1 (± 0.4)	157.2 (± 0.4)	0.1 (0.7)	142.9 (± 0.6)
1250	7	180	157.5 (± 0.9)	157.5 (± 0.6)	0.2 (0.5)	156.6 (± 0.9)
1500	2	180	147.9 (± 7.5)	148.1 (± 7.8)	0.3 (0.5)	139.5 (± 10.2)

Across all frequencies examined, the SPLs measured at the centre of the Test-Tank (TC) of the in-phase condition (0°) were distinctly higher than those in the out-of-phase condition (180°) (Table 1; Fig. 4). At the same time, no differences were observed between the SPLs in the in-phase and out-of-phase conditions at the reference positions, RT and RB across all frequencies and experimental trials (Table 1; Additional File 1: Sheet1).

Clear differences in SPLs between the positions RT/RB and TC were observed in the in-phase and out-of-phase conditions, except for one case, at 1250 Hz in the out-of-phase condition, where the difference in SPLs was only 0.9 dB (Table 1; Fig. 4b). Measurements regarding the positioning of the hydrophone head in relation to the centre of the tank and the tube wall (Fig. 4c2) indicate that the precision and calibration of the shakers were unaffected by whether the hydrophone head was situated at the centre of the water column or aligned with the wall level (Fig. 4c1; Additional File 1: Sheet1). Therefore, the positioning, where the front end of the hydrophone was aligned with the tube wall, was suitable for experimental measurements. Ambient noise measurements at both reference positions RT and RB in the Img-Tank gave an SPL of 118.0 dB re 1μPa (RT) and 118.5 dB re 1μPa (RB).

### Motion of auditory structures depends on maximum and minimum sound pressure

**Fig. 5:**
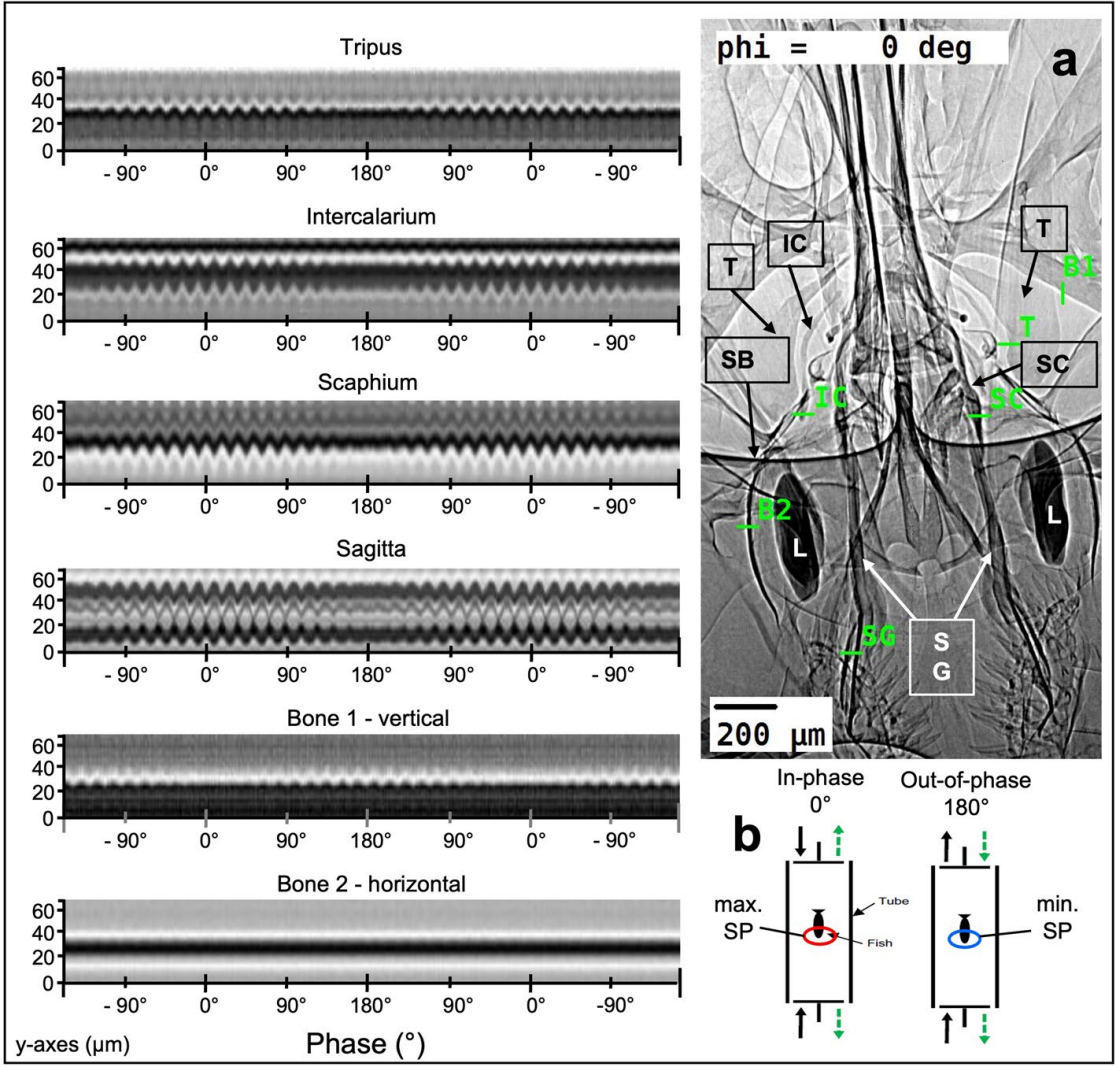
Visual representation of the movements of hearing structures during sound pressure and sound-induced particle motion. In the left column, motions of different auditory structures in *Kryptopterus vitreolus* are visualised by plotting the intensity profiles (*y*-axis) for line cuts through the respective structures in the radiographic image, as indicated in panel (**a**), as a function of the beating phase (*x*-axis). For clarity, two (identical) retrospectively averaged beating cycles are plotted in succession. (*y*-axes extent = 68.75 µm). **a** shows a radiographic image of the specimen at 0° phase. The locations of the line profiles through the individual structures, indicated by their abbreviations, are marked with green lines. The video corresponding to the figure is shown as Additional File 2: Video S1. **b** showing an illustration of both phases, namely the condition of maximum (in-phase) and minimum (out-of-phase) sound pressure (SP) within the tube centre during experiments (modified after Maiditsch et al. [23]). B1 bone 1, B2 bone 2, IC intercalarium, L lagena, SB swim bladder, SC scaphium, SG sagitta, T tripus

In Fig. 5 (and Additional File 2: Video S1), we present radiographic evidence for the response to maximum and minimum sound pressure fluctuations in the same experiment conducted with *K. vitreolus* (glass catfish, Otophysa), where one can observe the responses of the tripus, intercalarium, scaphium (Weberian ossicles) and the sagitta (= saccular otolith) to the two different sound conditions. During the 0° in-phase condition, the anterior swim bladder walls begin to oscillate, and the tripus moves along the horizontal line, as well as the intercalarium and scaphium, up to the sagitta; the latter tilts laterally and thus shows a rotational motion (Additional File 2: Video S1). The lateral motion becomes apparent in the temporal evolution of the intensity profiles along a lateral cut direction, exhibiting clear oscillations in the presence of motion (Fig. 5, left). In contrast, during the out-of-phase condition (180°) the temporal variation in the intensity profiles disappear, hence no clear motion of the hearing structures is observed. In all, the tripus, intercalarium, scaphium, and sagitta exhibit a substantial motion in response to sound pressure. This does not imply that no movements occur (otoliths) during minimum sound pressure; rather, movements are much smaller than in the maximum sound-pressure regime and thus too small to be visible with the given spatial resolution.

To illustrate the differences in movement between the Weberian ossicles and the sagittae compared to other structures, we measured intensity changes through two static bones (Fig. 5: Bone 1, Bone 2). Bone 1 represents the vertical (rostrocaudal) axis and Bone 2 the horizontal (mediolateral) axis. Along the horizontal axis, Bone 2 shows no motion under any condition, unlike the sound-induced motion of the auditory structures in the in-phase condition. In contrast, along the vertical axis, Bone 1 exhibits a minute up and down movement during the 180° condition, but not in the 0° condition. This suggests a global motion of the entire specimen in response to coherent particle motion in the water. Note that the horizontal intensity profiles through the individual auditory structures are not sensitive to this global vertical motion. Together, these observations reveal a collective motion response of the auditory system to the varying conditions imposed, rather than a random wobble of the structures within the sound field.

## Discussion

The results demonstrate that our advanced miniature standing wave tube provides a new avenue for non-invasive, high-resolution, time-resolved synchrotron-radiation-based tomography of sensory systems to contribute to a more comprehensive understanding of fish auditory mechanisms. Due to its shape and light materials, the current tank design does not represent an ideal standing-wave tube. However, the improvements made in this remodelled setup have effectively addressed specific shortcomings observed in previous designs, resulting in a reliable and optimised setup for our measurements. In previous studies, the reliability of achieving the desired acoustic conditions was limited. Schulz-Mirbach et al. [28] were restricted to observing the motion of fish hearing structures in 2D radiographic experiments at a fixed orientation and frequencies up to 200 Hz. Maiditsch et al. [23] examined the 3D motion of fish auditory structures; however, their test frequencies were limited to 450 Hz and establishing clear differences between sound pressure and particle motion was challenging. Considering that the fish species currently studied (otophysan) possess specialised auditory structures (Weberian apparatus) for detecting the pressure component of sound and responding to frequencies between 500 Hz and 3 kHz [14, 22], producing sound stimuli at higher frequencies becomes increasingly important. Consequently, our objective was to extend the frequency range and the remodelled setup made it possible to measure up to 2 kHz.

Furthermore, we were able to reduce SPLs, which were relatively high in previous studies [27]. In the closed setup (no hydrophones [23]), the SPL at 450 Hz was 183.2 dB re. 1 μPa. In contrast, with the new setup, we have successfully reduced the SPL at 450 Hz to an average of 162.4 dB re. 1 μPa at the centre of the tube (Table 1). Nevertheless, the SPLs are still considerably higher than the auditory thresholds measured in otophysan fish (below 80 dB [14]). It is important to note that we are imaging the movements of fish hearing structures within a range of a few micrometres, which represent natural motion. Due to limitations in the imaging procedure, it is currently not possible to visualise natural hearing structure movements at a nanometer scale under natural SPLs. Therefore, reducing the SPLs to a lower range presents technical challenges with the available imaging equipment. However, as the motion of the hearing structures, such as that observed in the glass catfish and previous studies, exhibited a consistent and reproducible pattern, we conclude that these observed patterns may also apply to biologically relevant SPLs [23, 28]. Certainly, this will require further studies to investigate the dependency on SPL.

The hydrophone measurements and the 2D radiograph recording of *K. vitreolus* (Additional File 2: Video S1) demonstrate that the shaker adjustments effectively generate maximum or minimum sound pressure. The high-resolution in situ images of the auditory structures of the glass catfish (Fig. 5) show the possibility of directly manipulating the sound field within the tube. Our data clearly indicate that in otophysan fishes, the motion of auditory structures depends more on sound pressure. As the impact of varying shapes and dimensions of the otoliths, the Weberian ossicles, and the swim bladder on otolith motion and on the structures’ motion patterns is still elusive, our approach enables further studies to compare motion patterns of species differing in the morphology of their auditory structures.

## Conclusions

Our data demonstrate that this setup works effectively, allowing us to obtain 4D tomographic images of the motion patterns of fish hearing structures when exposed to sound. We successfully established a controlled standing wave tube-like setup tailored for this specific purpose. Over 500 experiments were conducted at various stimulation frequencies and amplitudes on several distinct specimens, demonstrating consistent results and emphasising the reproducibility of the data with this configuration. Initial insights can be found in Qu et al. [35]. The current setup can now provide critical evidence to test previously unanswered key hypotheses in fish bioacoustics. For instance, Chranilov [36, 37] proposed a debated hypothesis on the functional morphology of the Weberian apparatus. His anatomical study of cypriniform and siluriform species revealed shape differences in the posterior region of the tripus linked to different degrees of swim bladder encapsulation. Based on the morphological differences, he suggested two different sound pressure transduction mechanisms, which still await experimental testing. Such experiments are now possible with our setup, which will also enable the examination of other small aquatic organisms (e.g. crustaceans) and can be adapted or modified accordingly depending on the experiment.

## Supplementary Information

Additional file 1: Excel sheets with data collected during the experiments, organised into separate sheets. Sheet1_Experimental_Data: summarized data of analyses and tables. Tables of Test-Tank measurements at different frequencies, with hydrophones placed in two different positions and experimental tests done using the Img-Tank and the Test-Tank. Sheet2_Raw_Data_beamline_exp: raw data used for analysis, categorized by frequency, phases (0° & 180°) and positions (RT, RB & TC). Sheet3_Raw_Data_SiG_setting: raw data including individual signal generator settings and measurements with both shakers operating simultaneously measured with Img-Tank, categorized by frequency, phases (0°& 180°) and positions (RT, RB & TC).

Additional file 2: Video S1: Real-time video corresponding to Figure 6. A 2D radiographical evidence for the presence of minimum sound pressure (sound-induced particle motion 180°) and maximum sound pressure (0°) during the same experiment, conducted on *Kryptopterus vitreolus*. Responses of the tripus, intercalarium, scaphium (Weberian Apparatus) and the sagitta to alternating levels of sound pressure during a beat can be observed (for details see Method section: Different sound conditions within the tube). Different phases between the two shaker stimuli are shown by the value of phi. The green lines through the individual structures are marking the cut direction for measurements in Figure 5 and show the structures of interest (T – Tripus; IC – Intercalarium; SC – Scaphium; SG – Sagitta; B1 – bone 1; B2 – bone 2). It is recommended to watch the video in a continuous loop to closely follow the movement sequence.

## Data Availability

All data generated or analysed during this study are included in this article or listed as supplementary information files. The full set of technical drawings of our acoustic tank setup has been published as PDF and stp-files for open access online on Zenodo (10.5281/zenodo.16737258) [30].

## Abbreviations

B1: Static bone (stands for vertical motion)
B2: Static bone (stands for horizontal motion)
CH: Centre hydrophone
D: Diameter
ESRF: European Synchrotron Radiation Facility
EPS: External power supply
Img-Tank: Imaging tank with two openings
H1: Hydrophone located near the upper shaker
H2: Hydrophone located near the lower shaker
IC: Intercalarium
L: Lagenar otolith
PSI: Paul Scherrer Institute
PM: Particle motion
RB: Reference bottom
RT: Reference top
SLM: Sound level meter
SB: Swim bladder
SC: Scaphium
S.E.: Standard error
SG: Sagitta
SiG: Signal generator
SLS: Swiss Light Source
SPLs: Sound pressure levels
SP: Sound pressure
T: Tripus
TB: Test bottom
TC: Test centre
Test-Tank: Test Tank with five openings
TT: Test top
Vpp: Peak-to-peak voltage
